# American Veterinary Medical Association

**Published:** 1900-07

**Authors:** S. Stewart

**Affiliations:** Secretary


### SOCIETY PROCEEDINGS.

### AMERICAN VETERINARY MEDICAL ASSOCIATION.

DETROIT, MICHIGAN, SEPTEMBER 4, 5, 6, 1900.

THE details of the coming meeting of the Association are about completed, and it would seem that nothing was lacking to make this the banner meeting of the Association. The local Committee of Arrangements has made excellent and complete preparations to receive the veterinarians of the country and treat them most royally. And it is expected that the ladies will accompany the veterinarians, for special arrangements have been made for their entertainment.

*Headquarters.* The headquarters of the Association will be at the Russell House, corner of Woodward avenue and Cadillac Square. There are numerous hotels in the immediate vicinity of the Russell House, and the tastes of the guests may be easily suited in style and price. If members and visitors will communicate with Dr. S. Brenton, chairman of the local Committee of Arrangements, 121 W. Alexandrine avenue, Detroit, Mich., stating full particulars as to the accommodations they may wish all arrangements will be made in advance.

*Place of Meeting.* The sessions of the Association will be held in the large convention hall of the Russell House, corner of Woodward avenue and Cadillac Square.

Special meetings of the Finance Committee, Publication Committee, and Executive Committee will be held at the Russell House on Monday afternoon, September 3. The regular session of the Associaton will convene at 9 A.M., Tuesday, September 4. The Mayor, Hon. William C. Maybury, will deliver the address of welcome, the response to be made by Dr. W. Horace Hoskins.

The literary programme will include the following papers: "Labor, Rest and Confinement," by Dr. W. L. Williams, Ithaca, N. Y. "Relation of Veterinary Medicine to the Public Health," by Dr. Wm. Herbert Lowe, Paterson, N. J. "Psoroptes Communis, Equi and Bovis, in Montana and Northwest Territory," by Dr. M. E. Knowles, Helena, Mont. "Remote Effects of Drugs," by Dr. L. A. Merrillat, Chicago, Ill. "Examining and Licensing of Stallions for Stud Purposes," by Dr. J. I. Gibson, Denison, Iowa. "The Relation of Lymphatics to Meat Inspection," by Dr. Tait Butler, Indianapolis, Ind. "Notes on the Tuberculin Test in Wisconsin," by Dr. G. Ed. Leech, Milwaukee, Wis. "Antiseptic Therapeutics, by Dr. J. F. Winchester, Lawrence, Mass. "Rabies," by Dr. D. S. White, Columbus, Ohio. "Urinalysis in Veterinary Practice," by Dr. Pierre A. Fish, Ithaca, N. Y. "Veterinary Progress in Michigan," by Dr. William Jopling, Owosso, Mich. "Spavin, Its Etiology and Treatment," by Dr. W. J .Martin, Kankakee, Ill. "Inoculation Against Texas Fever," by J. W. Connoway, Columbia, Mo. "Snakes, Venoms and Antidotes," by Dr. E. M. Ranck, Philadelphia, Pa. "Obstacles to Enforcing Regulations Requiring Tuberculin Test in Interstate Cattle Traffic," by Dr. Austin Peters, Boston, Mass. "Practical Antiseptics," by Dr. G. A. Johnson, Sioux City, Iowa. "Control of Cattle Ticks," by Dr. Cooper Curtice, Raleigh, N. C. "Difficulties in the way of Municipal Meat Inspection in the South," by Dr. C. A. Cary, Auburn, Ala. "Experimental Tuberculosis, Human and Bovine, in Domestic Animals," by Dr. R. R. Dinwiddie, Fayetteville, Ark.

A series of surgical clinics will be held on Wednesday, Thursday, and Friday mornings at the Detroit Veterinary Sanitarium, rear 121 W. Alexandrine avenue. The clinics will begin promptly at 8 o'clock and continue two hours. A number of well-known surgeons expect to be present and demonstrate one or more operations each. The list of clinicians include: Dr. L. A. Merrillat, of the McKillip Veterinary College; Dr. W. L. La Baw, of the Veterinary Department of Harvard University; Dr. W. L. Williams of the New York State Veterinary College; Dr. A. H. Baker, of the Chicago Veterinary College; Dr. J. W. Adams, of the Veterinary Department of the University of Pennsylvania; Dr. H. D. Gill, of the New York-American Veterinary College; Dr. R. C. Moore, of the Kansas City Veterinary College. A large variety of cases requiring surgical interference will be provided by local veterinarians, the kinds of cases which practitioners are often called upon to treat, and which frequently prove troublesome, such as fibroma of the elbow, tumors of head and face, roaring, crooked tail, lameness due to diseases of the extremities, etc.

The following special operations have been promised: Ridgling castration under antiseptic precautions, with expectation of healing without suppuration, by Dr. W. L. Williams. Arytenoideraphy for cure of roaring, by Dr. L. A. Merrillat. Operation for tumor of elbow (shoe-boil), by Dr. W. L. La Baw. Immediate and permanent closure of alveolar opening into maxillary sinus following removal of tooth, by Dr. R. C. Moore.

### PROGRAMME OF ENTERTAINMENT.

Tuesday, September 4, 1900. Morning: Visit to Convention Hall to witness the opening exercises. Afternoon: Trolley ride to Palmer Park and visit to ex-Senator Palmer's celebrated log cabin.

Wednesday, September 5, 1900. Morning: Visit to the Detroit Museum. Afternoon: Ladies will be entertained at luncheon by Mrs. S. Brenton, at her residence, 121 W. Alexandrine avenue.

Thursday, September 6, 1900. Morning: Carriage ride about the city, visiting Belle Isle Park and Waterworks Park. Evening: Theatre party.

Friday, September 7, 1900: Visit to Parke, Davis & Co.'s Laboratory, where a light luncheon will be served; from there the trolley or boat will be taken to the Ste. Claire Flats, where a fish and frog supper will be tendered all the visitors by the local association.

The railroads have granted the usual excursion rate of full fare for the going trip and one-third fare for the return, on the certificate plan. This rate ought to insure a large attendance from the large area of which Detroit is the centre. Eastern veterinarians who have enjoyed a most wonderful increase in practice, and are wondering how they can leave their business, should remember it will be profitable to themselves and their patrons to gather with veterinarians from all parts of America to compare notes and learn from each others' experiences.

Veterinarians living in the vicinity of New York City, Philadelphia, Baltimore, and Washington can reach Detroit as quickly and as cheap as those living in the Missouri River Valley, and they should not hold it as too far for them to go to this meeting of the A. V. M. A.

S. STEWART,
Secretary.

